# Beyond Morphology: Molecular Evidence Supporting a Clonal Relationship Between Pancreatic Undifferentiated Carcinoma with Osteoclast-like Giant Cells (UCOGC) and Conventional Pancreatic Ductal Adenocarcinoma (PDAC)

**DOI:** 10.3390/cancers18182967

**Published:** 2026-09-14

**Authors:** Esmeralda Celia Marginean, Dilshad Dhaliwal, Kevin E. Fisher, Sonalben Italiya, Alis Dema

**Affiliations:** 1 Department of Pathology, Baylor St. Luke’s Hospital, 6720 Bertner Ave, Houston, TX 77030, USA; celia.marginean@bcm.edu (E.C.M.); dilshad.dhaliwal@yale.edu (D.D.); kefisher@texaschildrens.org (K.E.F.); sonalben.italiya@bcm.edu (S.I.); 2 Department of Anatomic Pathology, “Victor Babeș” University of Medicine and Pharmacy, Eftimie Murgu Square, No. 2, 300041 Timisoara, Romania; 3 Anapatmol Research Center, “Victor Babes” University of Medicine and Pharmacy, Eftimie Murgu Square, No. 2, 300041 Timisoara, Romania; 4 Department of Pathology, Texas Children’s Hospital, Houston, TX 77030, USA; 5 “Pius Brinzeu” Emergency County Hospital, Str. Liviu Rebreanu, No. 156, 300723 Timisoara, Romania

**Keywords:** pancreas, undifferentiated carcinoma with osteoclast-like giant cells (UCOGC), PDAC, next-generation sequencing

## Abstract

**Background:** Undifferentiated carcinoma with osteoclast-like giant cells (UCOGC) is a rare pancreatic malignancy with striking morphology. Its prognosis appears more heterogeneous than its high-grade histology would suggest. Larger clinicopathologic and population-based studies suggest that UCOGC may behave more favorably than conventional PDAC. However, the prognostic impact of an associated PDAC component remains unresolved. **Methods:** Six cases of pancreatic UCOGC, diagnosed between 2019 and 2024, were retrospectively identified and selected for immunohistochemical and molecular analysis. We retrieved available clinical, radiologic, and follow-up data from electronic medical records. We performed targeted next-generation sequencing on individually macro-dissected UCOGC and PDAC components. **Results:** The cohort included four females and two males, with a median age of 68.5 years. All tumors were associated with conventional PDAC, and, in this series, only two cases were correctly diagnosed at biopsy; others were interpreted as PDAC or sarcomatoid carcinoma, with a definitive UCOGC diagnosis made only at resection. Immunoreactivity for cytokeratin was observed in the epithelial component, while the undifferentiated and giant cell components showed vimentin and CD68 immunoreactivity, respectively. NGS revealed co-mutations in *TP53* and *KRAS* in 4 cases. Pathogenic mutations in *APC*, *BRCA2*, *BRAF*, *GNAS*, *PIK3CA*, *RB1*, *SMAD4*, and *CDKN2A/B* loss were also detected. concordant pathogenic variants Paired analysis of macro-dissected components from the same tumor (n = 4) demonstrated concordant pathogenic variants, providing supportive evidence of a shared clonal relationship. Two patients received neoadjuvant chemotherapy, and systemic therapy was administered in five patients. Three patients were alive during the last follow-up, including two long-term survivors. **Conclusions:** Pancreatic UCOGC shares key genetic alterations with PDAC, and paired molecular analysis provides supportive evidence of a shared clonal relationship between the UCOGC and ductal components. The variable clinical outcomes observed in this small series are descriptive and hypothesis-generating and require validation in larger cohorts.

## Simple Summary

UCOGC is a rare pancreatic cancer with distinctive histologic features and variable clinical behavior. We reviewed six cases diagnosed between 2019 and 2024, all associated with conventional pancreatic ductal adenocarcinoma (PDAC). In this series, only two cases were correctly identified on biopsy, highlighting both the diagnostic difficulty of this tumor and the potential for sampling limitations, particularly when the biopsy does not capture both components. Immunohistochemistry on resected tumors showed expected staining patterns in the epithelial, undifferentiated, and giant cell components. Molecular testing identified frequent KRAS and TP53 mutations, along with alterations in several other cancer-related genes. In four tumors, separate analysis of the UCOGC and PDAC components demonstrated mutations, concordant pathogenic variants, supporting the possibility of a shared clonal origin. Three patients were alive at last follow-up, including two long-term survivors. These findings suggest that UCOGC is molecularly related to PDAC, that its clinical course can vary considerably, and that the presence of an associated PDAC component does not necessarily indicate a poor prognosis.

## 1. Introduction

Pancreatic cancer ranks as the sixth leading cause of cancer-related mortality worldwide, accounting for approximately 467,005 deaths in 2022 [1,2]. Despite advances in oncologic care, the overall 5-year relative survival rate remains dismal at 13% in the United States, placing it among the malignancies with the poorest prognoses [3]. Alarmingly, the mortality rate of pancreatic cancer is projected to surpass breast and colorectal cancer by 2030 [4].

Approximately 90% of pancreatic tumors are of exocrine origin, with pancreatic ductal adenocarcinoma (PDAC)—an infiltrating gland-forming carcinoma—representing the predominant subtype. In contrast lies a rare and morphologically distinct subset of pancreatic neoplasms, collectively termed undifferentiated pancreatic carcinomas, which lack glandular differentiation and often display prominent histiocytic and osteoclast-like giant cell infiltration [5]. These tumors are typically hypercellular with minimal stroma and poor cellular cohesion [6].

According to the World Health Organization (WHO) classification, undifferentiated pancreatic carcinomas encompass several histologic variants, including undifferentiated carcinoma with osteoclast-like giant cells (UCOGC), rhabdoid, and sarcomatoid types, as well as mixed forms [7]. Among them, UCOGC is exceedingly rare, constituting less than 1% of all pancreatic carcinomas [8]. Histologically, UCOGC is characterized by a biphasic pattern—pleomorphic neoplastic mononuclear cells intermingled with non-neoplastic multinucleated osteoclast-like giant cells. Based on cytologic composition, it can be subclassified into osteoclastic, pleomorphic, or mixed subtypes [7]. UCOGC typically presents in the sixth to seventh decades of life, with no clear sex predilection [9,10].

At the molecular level, UCOGC shares striking similarities with PDAC. Luchini et al. demonstrated recurrent alterations in classical pancreatic oncogenes and tumor suppressor genes—*KRAS*, *CDKN2A*, *TP53*, and *SMAD4*—in UCOGC, supporting its classification as a morphologic and molecular variant of PDAC [5]. Similarly, Hrudka et al. identified a comparable mutational spectrum in next-generation sequencing of UCOGC specimens, reinforcing the concept that this tumor may represent a dedifferentiated form of PDAC rather than a distinct entity [11]. Given its rarity and histologic heterogeneity, UCOGC remains underrecognized and poorly understood.

The objective of this study is to characterize the molecular alterations, histopathologic features, immunophenotypic profile, and clinical behavior of undifferentiated carcinoma with osteoclast-like giant cells of the pancreas, thereby enhancing current understanding of its pathogenesis, diagnostic challenges, and potential therapeutic implications. This study was approved by the Institutional Review Board (IRB) of Baylor College of Medicine, Houston, Texas, and all procedures were conducted in accordance with institutional guidelines. Due to the retrospective nature of the study, the requirement for informed consent was waived.

## 2. Materials and Methods

### 2.1. Case Selection

We searched our institution’s pathology archives for undifferentiated carcinoma with osteoclast-like giant cells (UCOGC) of the pancreas diagnosed between 2019 and 2024. Pathologists with expertise in gastrointestinal and pancreatic pathology retrieved the glass slides of the identified cases and confirmed them histologically on surgical resection specimens. For each case, tumor morphology was assessed on hematoxylin and eosin (H&E) stained sections, with particular attention to the presence and distribution of pleomorphic mononuclear tumor cells, non-neoplastic multinucleated osteoclast-like giant cells, spindle cell areas, and any associated epithelial component. Representative formalin-fixed, paraffin-embedded (FFPE) tissue blocks were selected for immunohistochemical and molecular analyses. For cases sampled preoperatively by endoscopic ultrasound, tissue was acquired using a 22-gauge Boston Scientific (300 Boston Scientific Way, Marlborough, MA, USA). Acquire fine-needle biopsy needle to obtain adequate core tissue for histopathologic evaluation and, when indicated, ancillary molecular studies. We retrieved available clinical, radiologic, and follow-up data from electronic medical records.

### 2.2. Immunohistochemistry (IHC)

We performed IHC on 4 µm FFPE sections using the Dako Autostainer Link 48 platform (Agilent Technologies Inc., 5301 Stevens Creek Blvd, Santa Clara, CA, USA). Primary antibodies included cytokeratin AE1/AE3 (EMD Millipore Corporation 400 Summit Drive Burlington, MA, USA), CK7 (Ventana; Roche-clone SP52;Ventana Medical Systems, Inc. 1910 E. Innovation Park Drive, Tucson, AZ, USA), CK19 (Cell Marque, clone A53-B/A2.26, Cell Marque Corporation 6600 Sierra College Blvd Rocklin, CA, USA), vimentin (Ventana; clone V9, Ventana Medical Systems, Inc. 1910 E. Innovation Park Drive, Tucson, AZ, USA), CD68 (Ventana; clone SP251v18, Ventana Medical Systems, Inc. 1910 E. Innovation Park Drive, Tucson, AZ, USA), and p53 (Ventana; clone VO-7, Ventana Medical Systems, Inc. 1910 E. Innovation Park Drive, Tucson, AZ, USA), among others, as appropriate. We followed standard antigen retrieval and staining protocols per manufacturer recommendations. Slides were counterstained with hematoxylin, dehydrated, and mounted with a xylene-based medium. Two pathologists independently reviewed all IHC slides: one GI-trained senior pathologist and one trainee (ECM, DD). We recorded immunoreactivity for each cell type.

### 2.3. Targeted Next-Generation Sequencing (NGS)

For molecular profiling, tumor-rich areas were macro-dissected from FFPE blocks to separately analyze spindle/giant cell and PDAC components when both were present. DNA extraction was performed from each component using the QIAamp FFPE Tissue Kit (Qiagen, Hilden, Germany) according to the manufacturer’s protocol. DNA quantity and quality were assessed using a Qubit 3 Fluorometer (Thermo Fisher Scientific, Wilmington, DE, USA). We performed a targeted, custom-designed tumor-only NGS assay for coding and splicing regions of 2867 exons in 152 genes, as described previously [12]. Matched germline analysis was not performed. Briefly, barcoded libraries were constructed from genomic DNA with the KAPA Biosystems HyperPlus kit followed by capture hybridization-based target enrichment using a custom-designed Roche NimbleGen SeqCap Target Enrichment probe set and sequencing on the Illumina MiSeq System. FASTQ files were aligned to the GRCh37 (hg19) reference human genome using Burrows–Wheeler Aligner (BWA) and NextGENe v2.4.1.2. Variant calling was performed using NextGENe v2.4.1.2 and Platypus v0.8.1 and variants were annotated with Variant Effect Predictor to produce a merged annotated variant list. Average read depths of 250× for each interrogated base were obtained, and a 5.0% variant allele frequency (VAF) cut-off was used prior to variant interpretation. Presumed somatic variants were interpreted and classified as variants of established/potential (Tier I/II) or uncertain (Tier III) clinical significance. Variants that met diagnostic, prognostic, or therapeutic significance criteria per published somatic variant classification criteria [13] were considered Tier I/II. All other variants that did not meet Tier I/II criteria were considered Tier III.

### 2.4. Data Analysis

We summarized clinicopathologic and molecular findings descriptively. We assessed correlations between morphologic patterns, immunoprofile, and molecular alterations to identify potential pathogenetic relationships between UCOGC and PDAC components.

## 3. Results

We identified six patients with undifferentiated carcinoma with osteoclast-like giant cells (UCOGC) of the pancreas over the study period. The median age at diagnosis was 68.5 years (range, 60–74 years), and the cohort consisted of four females and two males. Tumors arose throughout the pancreas, involving the head (n = 3), neck (n = 1), tail (n = 1), and body/tail with peripancreatic extension (n = 1). The maximum tumor dimension ranged from 1.5 cm to 8.1 cm, with a median size of 4.2 cm, demonstrating substantial size variability at presentation.

### 3.1. Pathologic Features

**Figure 1:**
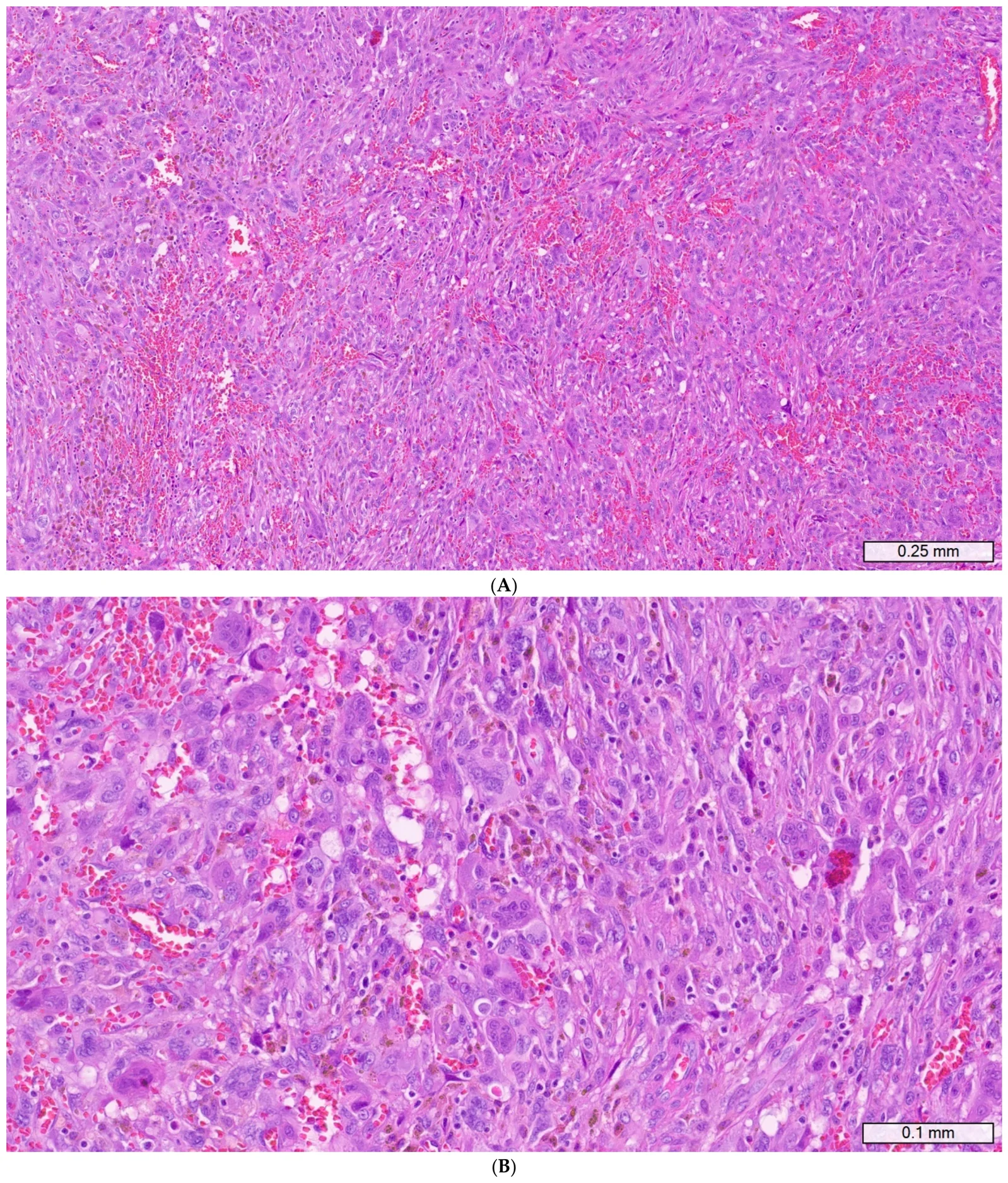
(**A**) Undifferentiated component, composed of fascicles of spindle cells with atypical nuclei, intermixed with a variable amount of osteoclast-like multinucleated giant cells and smaller mononuclear histiocytes. Hematoxylin and eosin stain (H&E). (**B**) Higher magnification highlighting abundant osteoclast-like multiloculated giant cells admixed with a few mononuclear histiocytes and spindle tumor cells. Hematoxylin and eosin stain (H&E).

Resection specimens from all six cases showed a biphasic tumor composed of a predominant undifferentiated (sarcomatoid) component, arranged in solid, fascicular, and focally storiform sheets of neoplastic spindle and epithelioid cells with moderate-to-marked nuclear pleomorphism and readily identifiable mitotic activity, admixed with mononuclear histiocytes and non-neoplastic osteoclast-like multinucleated giant cells (Figure 1A,B).

**Figure 2:**
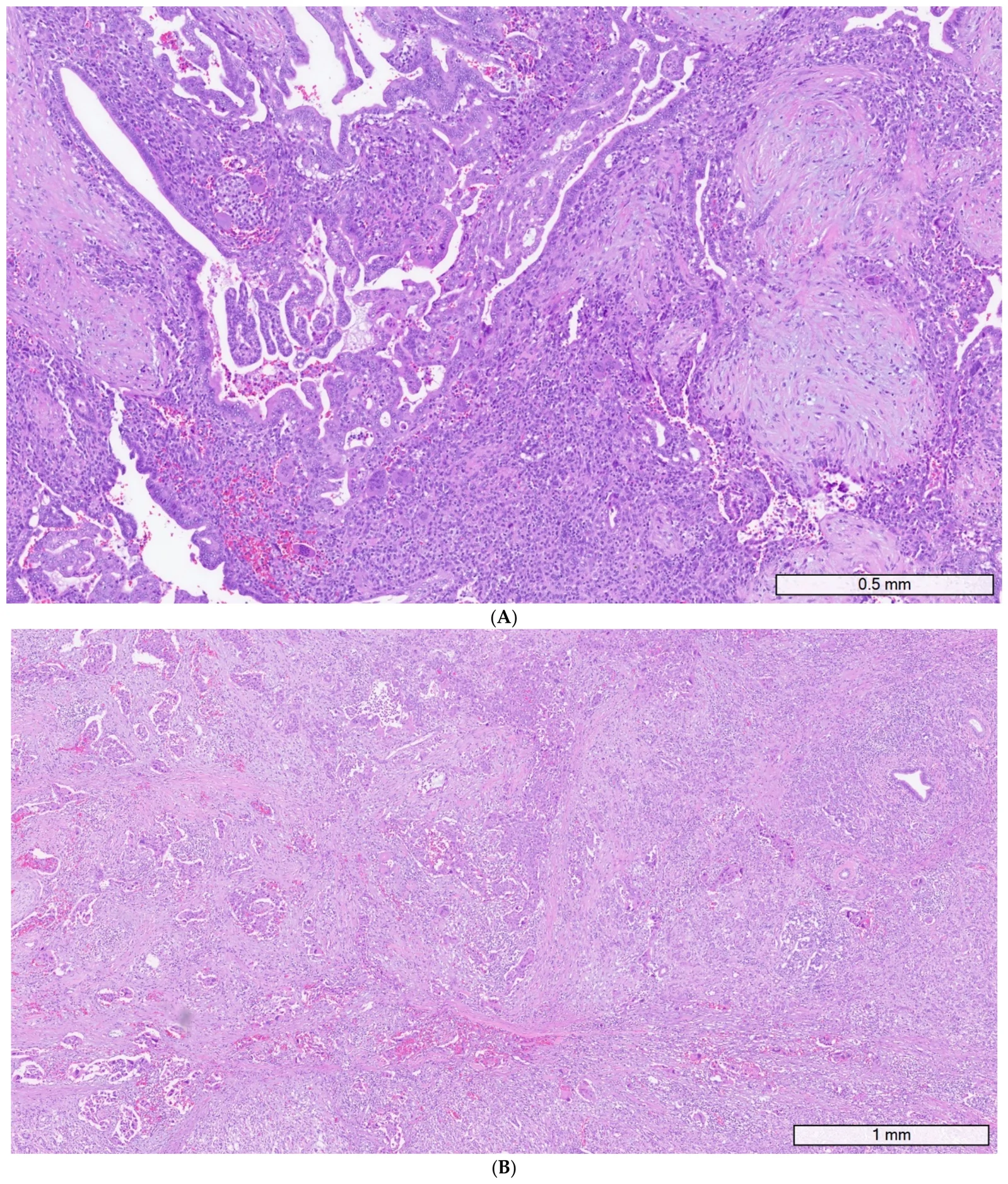

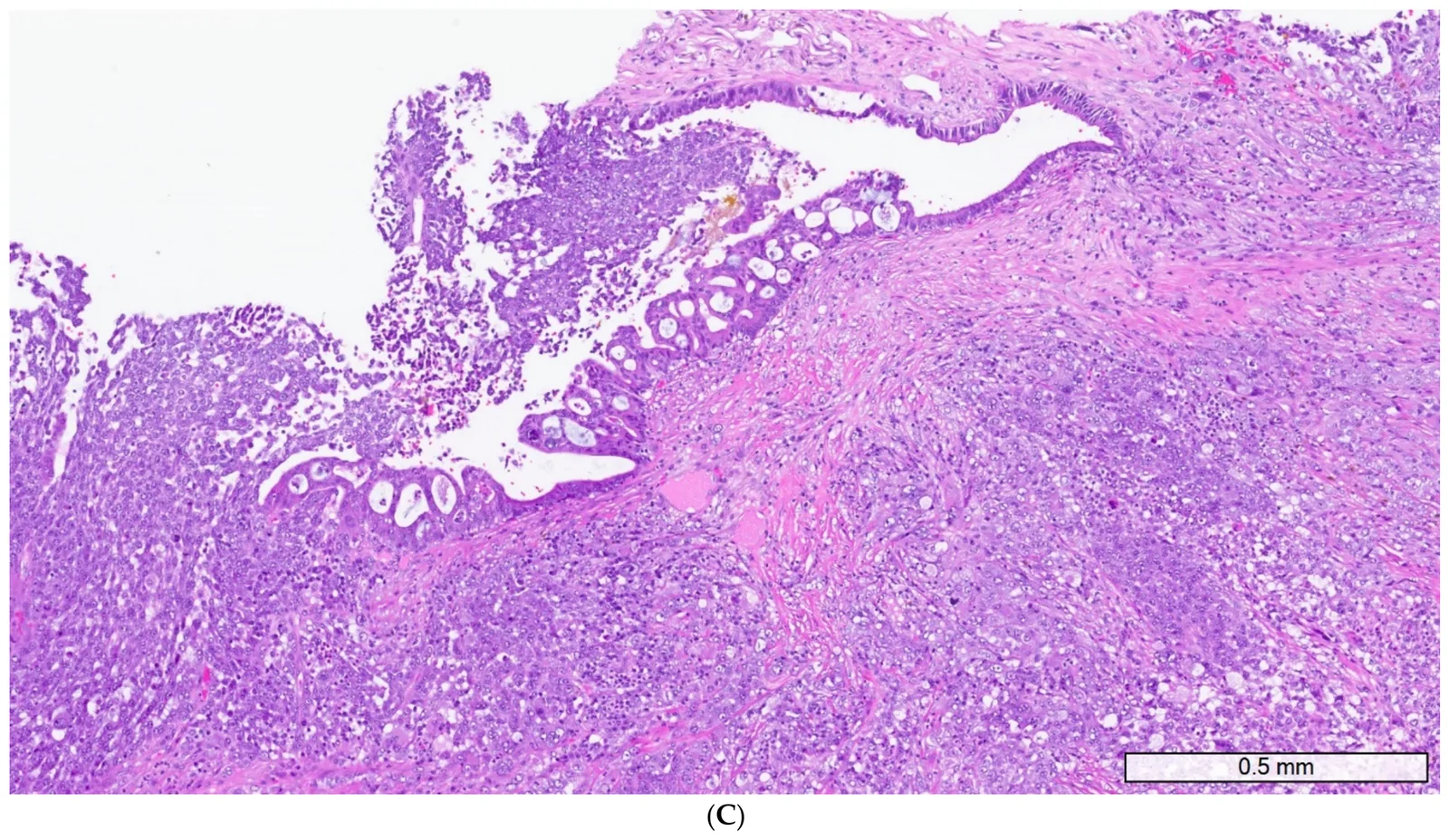
(**A**) This tumor showed a significant epithelial component, composed of conventional pancreatic ductal adenocarcinoma (PDAC), (upper left and lower left of the image), relatively well-delineated from the undifferentiated spindle cell component (lower right side of the image). Hematoxylin and eosin stain (H&E). (**B**) The epithelial component varied from very scant, with a few glands present at the periphery of the tumor, to almost half of the tumor, as shown in this image. The entire left side of the image shows conventional adenocarcinoma, with the spindle cell undifferentiated component in the upper right corner. Hematoxylin and eosin stain (H&E). (**C**) Two of the tumors showed an associated intraductal papillary mucinous neoplasm (IPMN) with high-grade dysplasia and associated invasive conventional adenocarcinoma, in addition to the undifferentiated component. IPMN is shown in the upper left of the image; the main pancreatic duct is markedly dilated, partially filled by a papillary proliferation with high-grade dysplasia. The remainder of the tumor in this image is composed entirely of the undifferentiated spindle cell component. Hematoxylin and eosin stain (H&E).

The relative proportion of osteoclast-like giant cells to mononuclear tumor cells varied considerably across the cohort, spanning the morphologic continuum recognized by the WHO classification, from giant cell-rich tumors more in keeping with the osteoclastic subtype (one case) to giant cell-sparse examples with a predominantly pleomorphic mononuclear population (one case); 4 tumors displayed overlapping, mixed features. This morphologic heterogeneity was mirrored by the associated epithelial component, which was identifiable in all six tumors (Figure 2A) but differed substantially in extent, ranging from a minimal, peripheral rim of glands in most cases to nearly half of the overall tumor volume in one case (Figure 2B). Two of the six tumors additionally harbored a prominent intraductal papillary mucinous neoplasm (IPMN) with high-grade dysplasia and an associated invasive conventional component (Figure 2C), further illustrating the range of ductal-lineage precursor lesions that may accompany UCOGC.

**Figure 3:**
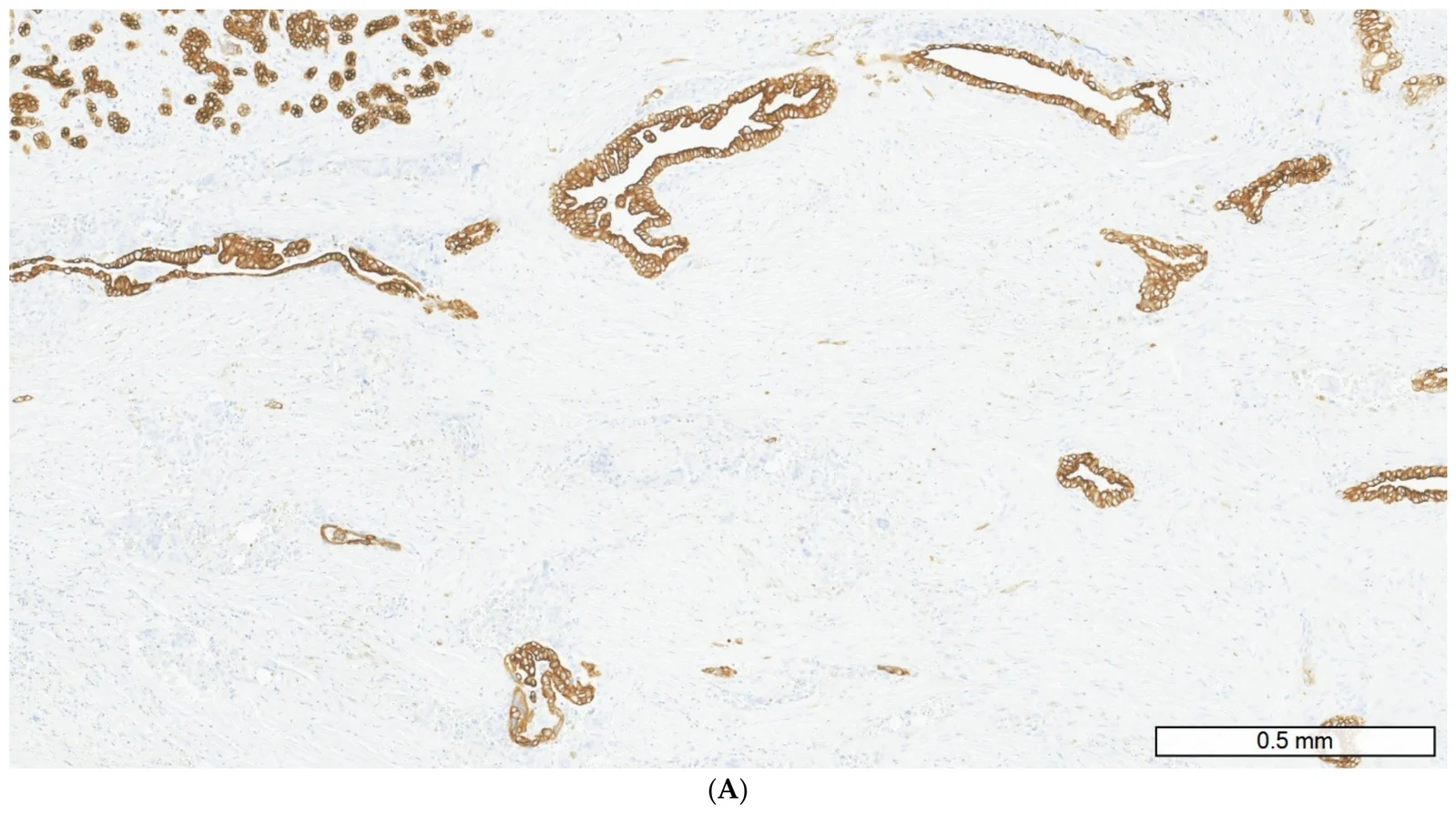

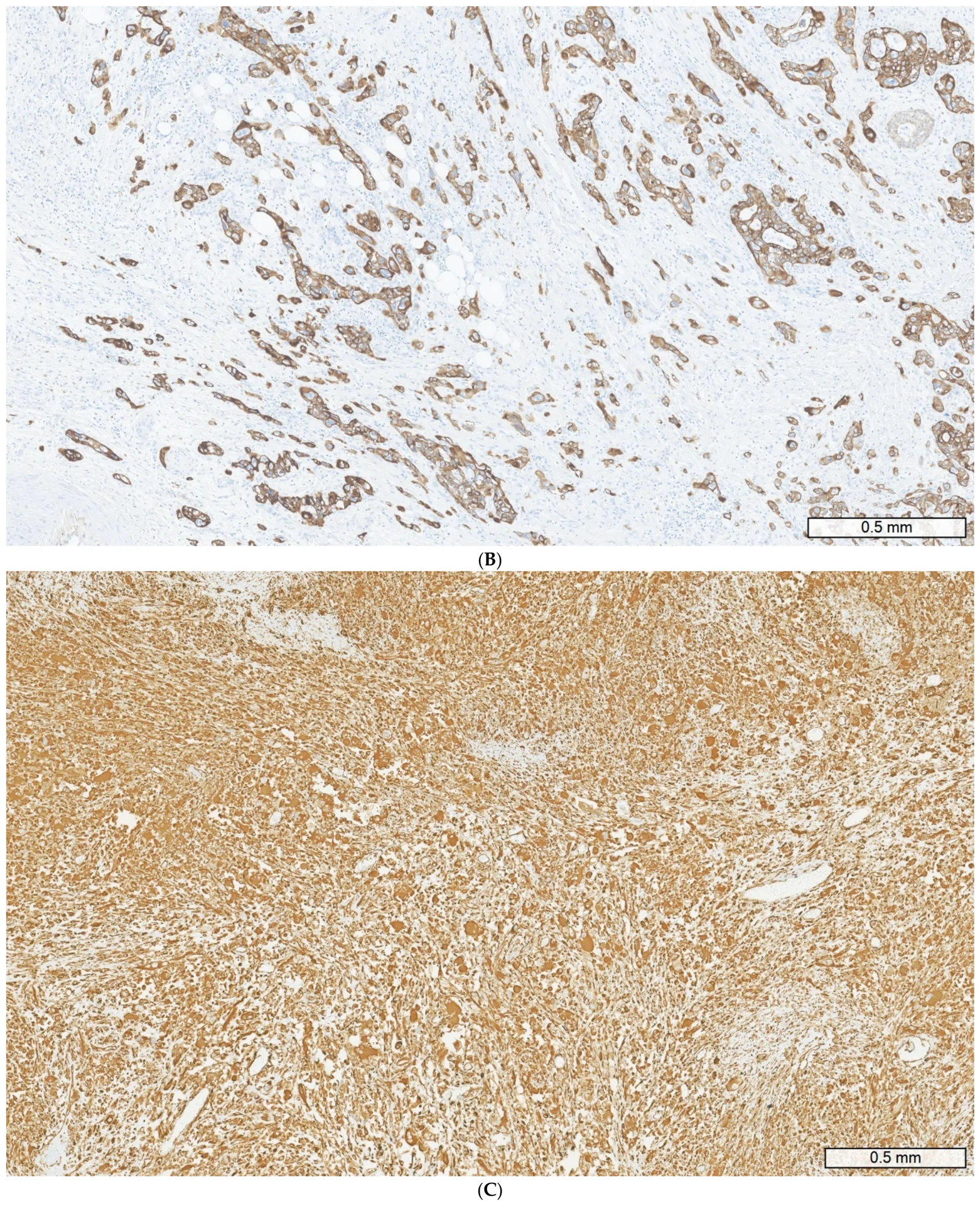

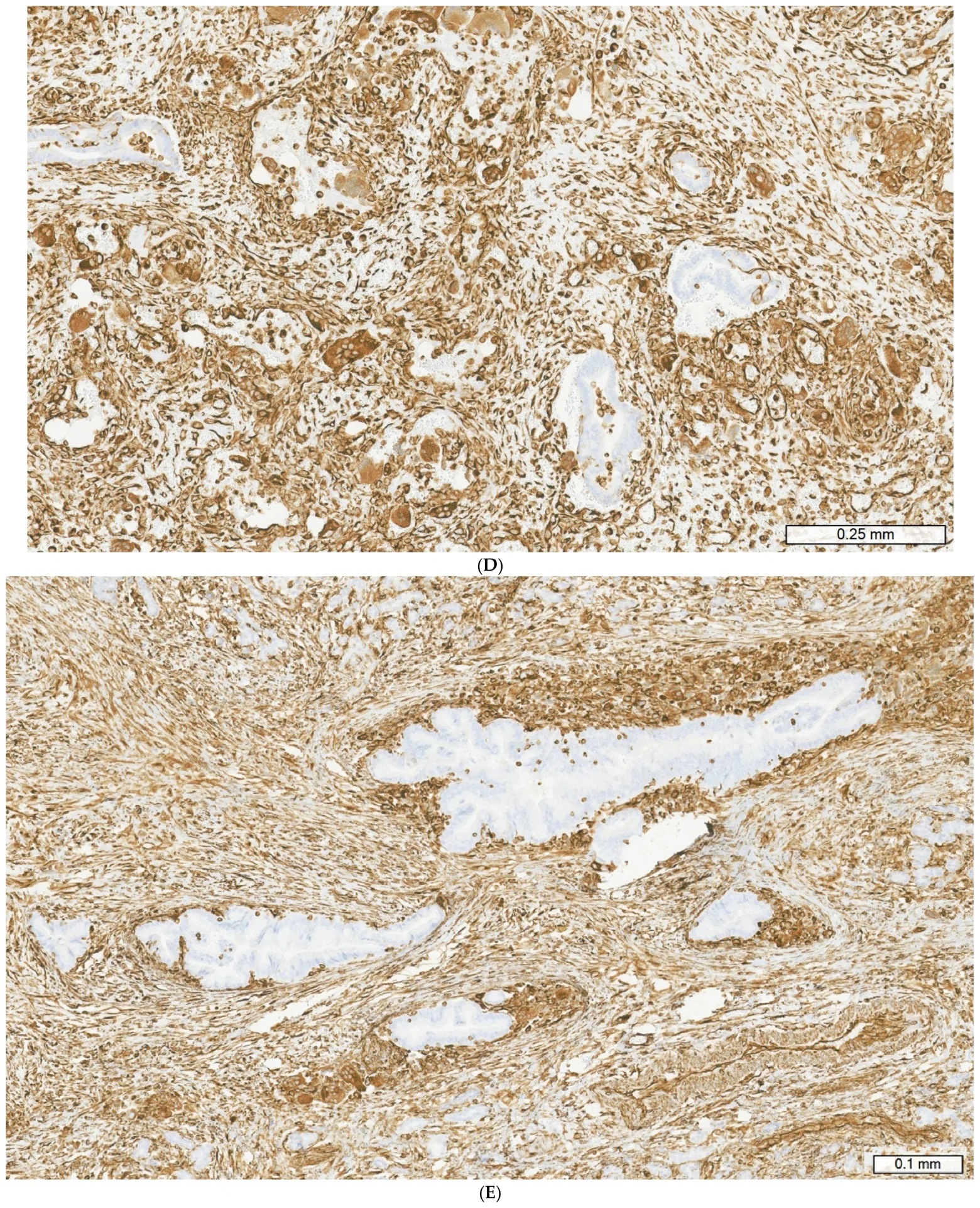

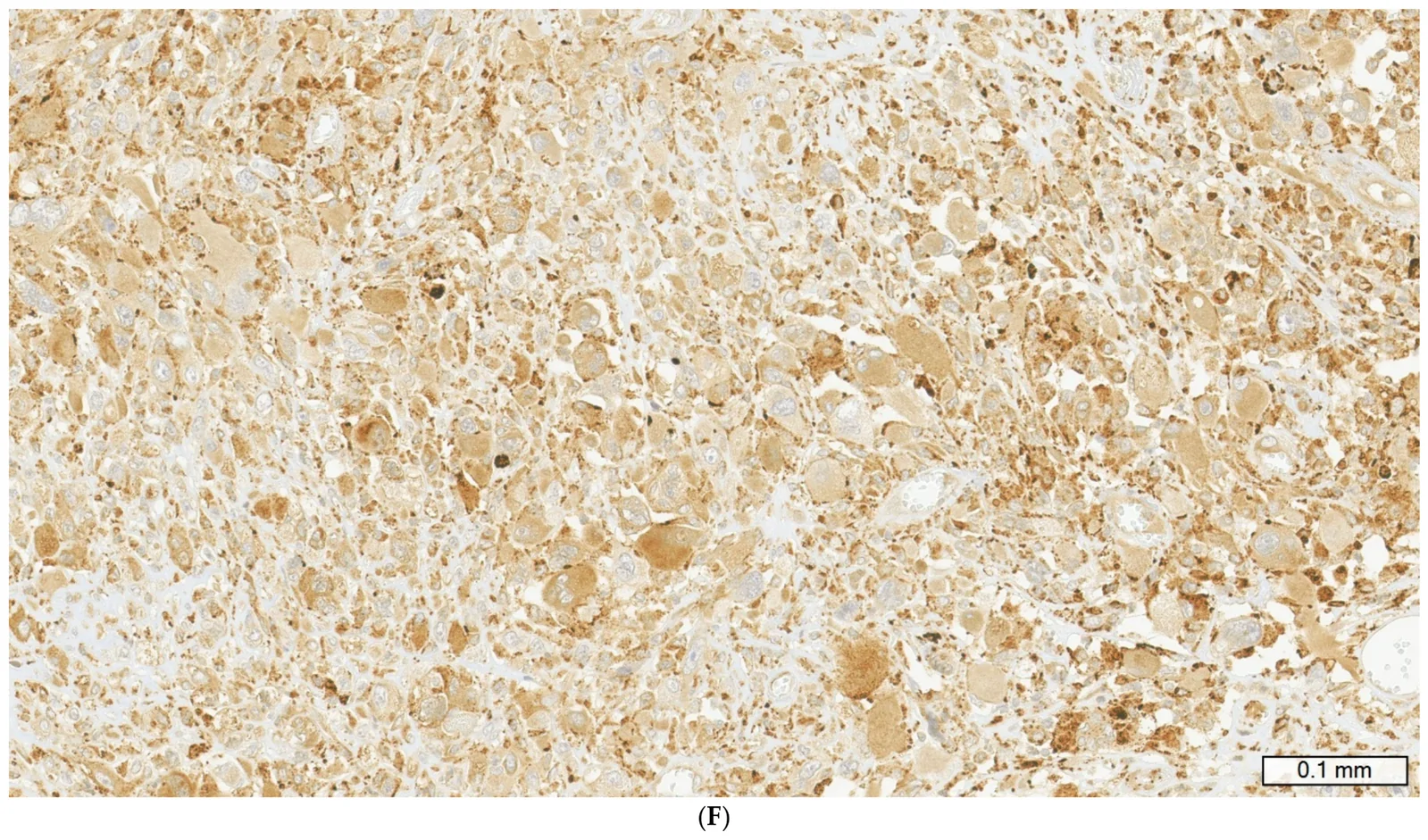
(**A**) Pancytokeratin immunohistochemical stain, highlighting the malignant epithelium, as well as benign ducts (upper left corner). PDAC component in this case is very scant, at the periphery of the tumor. The undifferentiated component is negative. (**B**) Pancytokeratin immunohistochemical stain showing a very large PDAC component in a different tumor. Again, the interspersed spindle cells are negative. (**C**) Vimentin immunohistochemical stain shows strong and diffuse immunoreactivity in the undifferentiated component, staining all the spindle cells, as well as the nontumoral osteoclast multinucleated giant cells and mononuclear histiocytes. (**D**) Vimentin immunohistochemical stain highlights the undifferentiated component, osteoclast multinucleated giant cells, and histiocytes, but is completely negative in the epithelial component (malignant glands in the lower right and upper left of image). (**E**) Higher magnification shows that PDAC epithelium is negative for vimentin immuhistochemical stain, while the spindle component is strongly and diffusely positive. (**F**) CD68 immunohistochemical stain highlights the osteoclast-like multinucleated giant cells and the mononuclear tumor cells, confirming their histiocytic origin.

These findings reinforce the notion that UCOGC may arise in association with recognizable ductal lineage lesions and that the conventional component may be small, peripheral, or easily missed in limited biopsy material. Across all six cases, immunohistochemistry consistently corroborated this biphasic morphology: pancytokeratin, CK7, and CK19 were expressed exclusively by the epithelial (PDAC and IPMN) component (Figure 3A,B), whereas vimentin was diffusely and consistently positive within the undifferentiated component (Figure 3C) and uniformly negative within the epithelial component in every case examined (Figure 3D,E). The osteoclast-like giant cells were strongly and diffusely positive for CD68 in all six cases, confirming their non-neoplastic histiocytic derivation (Figure 3F). This concordant immunophenotypic pattern across the cohort reinforced the distinction between the neoplastic mesenchymal-appearing and epithelial elements, regardless of the relative proportion of each component within an individual tumor.

We also compared additional adverse pathologic parameters across the cohort. Lymphovascular invasion (LVI) was identified in most cases (5 of 6), while perineural invasion (PNI) was present in half (3 of 6). Surgical margin status was similarly heterogeneous: three patients had R1 resections, most commonly involving the pancreatic neck or uncinate process, while the remaining three achieved negative (R0) margins. Regional lymph node metastases were identified in 3 of 6 cases (1 positive node in each), and distant metastatic disease, either at initial diagnosis or during subsequent follow-up, was documented in two patients. Given the small cohort, no conclusions can be drawn regarding associations between these adverse pathologic features, the relative proportions of UCOGC and epithelial components, and clinical outcome.

### 3.2. Molecular Profiling

Targeted next-generation sequencing revealed pathogenic Tier I/II variants in all samples. Four cases harbored *KRAS* and *TP53* co-mutations, and two cases showed loss-of-function alterations in *BRCA2*. Individual mutations in the *APC*, *BRAF*, *GNAS*, *PIK3CA*, *RB1*, and *SMAD4* genes, as well as CDKN2A/B loss, were also observed. Overall, these six pancreatic UCOGC cases showed a low-to-moderate apparent mutational burden, and no case demonstrated a clearly hypermutated profile based on the reported targeted molecular findings. Importantly, paired macro-dissected analysis was available in four tumors and demonstrated highly similar mutations in the UCOGC and conventional PDAC components, providing supportive evidence of a shared clonal origin. In two cases, comparative analysis was not feasible because of a limited PDAC component or insufficient nucleic acid for testing. Table 1 summarizes the clinically relevant molecular findings, while Supplementary Table S1 provides complete sequencing results, including variants of uncertain significance.

### 3.3. Treatment and Clinical Outcomes

Treatment exposure varied across the cohort, reflecting differences in disease extent, performance status, and patient preference (Table 1). All tumors were surgically resected. Two patients received neoadjuvant FOLFIRINOX (folinic acid, fluorouracil (5-FU), irinotecan, and oxaliplatin). Five of six patients (83.3%) received adjuvant therapy; when administered, this consisted of FOLFIRINOX (n = 3) or gemcitabine-based regimens (n = 2). One patient declined standard adjuvant therapy and received T-cell reinfusion.

Clinical follow-up was updated through 15 June 2026, which served as the study data cutoff for survival and outcome analyses. Three of the six patients (50%) were alive, including two long-term survivors who had achieved at least 71 months of disease-free survival (71 and 78 months, respectively). Notably, one long-term survivor (patient #3) had metastatic disease at presentation but remained radiographically stable for more than four years after adjuvant therapy. The remaining three patients (50%) died of disease. One patient died 1 month after diagnosis from disease-related complications, including lower-extremity swelling, ascites, and massive bilateral pulmonary emboli. The other two patients died 4 and 17 months after diagnosis, respectively, with metastatic disease.

In this small cohort, survival durations varied considerably, ranging from 1 to 78 months. Three patients died within 17 months, whereas three survived 56–78 months. Given the limited sample size, these observations are descriptive and should not be interpreted as evidence of distinct prognostic or biological subgroups. Rather, they illustrate the variability in clinical outcomes that may occur in UCOGC and warrant further evaluation in larger cohorts.

Because of the small sample size, we did not perform formal statistical modeling. Nevertheless, early mortality appeared to cluster with metastatic disease or extensive tumor invasion at diagnosis, whereas prolonged survival was not restricted to patients with favorable pathology. Long-term survivors included patients with nodal metastasis, lymphovascular invasion, perineural invasion, or positive margins, suggesting that adverse histologic features do not uniformly predict poor outcomes in UCOGC. Neoadjuvant or adjuvant therapy was common among longer survivors, but this cohort does not allow inference of therapeutic benefit. Treatment exposure was heterogeneous across patients, and no conclusions regarding treatment efficacy can be drawn from this cohort. Collectively, these findings support the concept that UCOGC is a biologically heterogeneous PDAC variant with a survival spectrum that may be wider than that of conventional PDAC.

## 4. Discussion

This study contributes to the evolving understanding of pancreatic undifferentiated carcinoma with osteoclast-like giant cells (UCOGC) by integrating detailed morphologic review, immunophenotyping, sequencing, and clinical follow-up in a single-institution cohort. Although the cohort is small, this limitation is inherent to UCOGC research and is consistent with the broader literature, which remains dominated by case reports, small institutional series, reviews, and retrospective database analyses [14,15,16,17]. However, the present study did not include a contemporaneous stage- or treatment-matched PDAC comparator; therefore, no direct conclusions about relative survival between UCOGC and conventional PDAC can be drawn [18,19].

The value of the present series lies not in statistical power but in its carefully annotated, component-specific pathologic and molecular assessment of a rare variant of pancreatic carcinoma.

The clinical behavior of UCOGC remains one of its most compelling and unresolved features. Diagnostic difficulty on limited biopsy material has also been described in larger clinicopathologic series and in studies of EUS-guided sampling, supporting the importance of adequate tissue acquisition and recognition of the heterogeneous tumor components [20]. Earlier studies, however, emphasized aggressive behavior and high early mortality, particularly in tumors with advanced disease or mixed anaplastic features [21,22]. Prior studies have reported variable outcomes in UCOGC, with some series suggesting longer survival than conventional PDAC [18,19]. However, differences in stage, resectability, treatment, patient selection, and follow-up make cross-study comparisons difficult. Because our study lacks a contemporaneous matched PDAC control group, our survival findings should be interpreted descriptively rather than as evidence of a prognostic difference between UCOGC and conventional PDAC.A practical message for diagnostic pathologists is that UCOGC is both recognizable and easy to underdiagnose on limited material. Several patients in our cohort were initially interpreted as having PDAC or sarcomatoid carcinoma on biopsy, reflecting the fact that small samples may capture only the malignant spindle/epithelioid component or only focal conventional ductal differentiation. Prior EUS/FNA-based series similarly emphasize that cytology and limited biopsies may be diagnostically challenging, but that identification of osteoclast-like giant cells, pleomorphic mononuclear tumor cells, and appropriate immunophenotypic separation can support the diagnosis [23,24]. Extensive sampling of resection specimens remains essential, particularly because associated PDAC, IPMN, or mucinous cystic neoplasm components have been repeatedly documented in UCOGC and may carry prognostic and pathogenetic significance [20,25,26,27]. Adequate tissue acquisition is particularly important in UCOGC because limited sampling may fail to capture its heterogeneous morphologic components and may also restrict ancillary molecular testing. Current European Society of Gastrointestinal Endoscopy recommendations emphasize optimization of EUS-guided tissue acquisition, including the use of fine-needle biopsy techniques for solid pancreatic lesions, to obtain diagnostically adequate tissue and support downstream ancillary analyses. The importance of obtaining a macroscopically adequate tissue core has also been demonstrated in prospective studies of EUS-guided fine-needle biopsy [28,29].

Our paired molecular findings further support a ductal lineage and common clonal origin. The shared *KRAS* and *TP53* alterations between morphologically distinct areas in our cases parallel prior molecular studies showing that UCOGC harbors the canonical PDAC driver profile, including *KRAS*, *CDKN2A*, *TP53*, and *SMAD4* alterations. This interpretation is also consistent with earlier histogenetic work demonstrating ductal differentiation, cytokeratin expression in the neoplastic mononuclear component, *KRAS* alterations, and direct evidence of ductal evolution in early lesions [30,31]. The non-neoplastic nature of the osteoclast-like giant cells is supported by their histiocytic phenotype, as established in classic immunohistochemical studies and mirrored in our CD68-positive giant cell population [21].

The morphologic divergence between UCOGC and conventional PDAC despite shared driver alterations suggests that additional biological mechanisms may contribute to the UCOGC phenotype. Prior studies have implicated features of the tumor microenvironment, including macrophage-rich inflammation and PD-L1 expression. These parameters were not systematically evaluated in the present study, and therefore their potential contribution to UCOGC biology remains hypothesis-generating and requires dedicated investigation. Epithelial–mesenchymal transition and chromatin remodeling may also contribute to the morphologic divergence of UCOGC from conventional PDAC. Mattiolo et al. showed differential activation of epithelial–mesenchymal transition programs in undifferentiated pancreatic carcinomas with and without osteoclast-like giant cells, implicating transcriptional programs rather than single pathognomonic mutations [32]. More broadly, SWI/SNF complex deficiency has been described in undifferentiated pancreatic carcinomas, particularly those with rhabdoid morphology, highlighting the need to distinguish UCOGC from other undifferentiated carcinoma subtypes rather than grouping all anaplastic pancreatic carcinomas together [33]. These observations support a model in which UCOGC is genetically PDAC-aligned but phenotypically shaped by transcriptional, epigenetic, and microenvironmental forces. These mechanisms were not directly assessed in our cohort and are discussed as biologically plausible hypotheses based on prior literature rather than conclusions derived from the present data.

Management of UCOGC is generally extrapolated from conventional PDAC because prospective treatment data are lacking. Population-based and retrospective studies have suggested potential benefit from surgery and systemic therapy. However, treatment exposure in our cohort was highly heterogeneous and influenced by factors including stage, resectability, performance status, and patient preference. Accordingly, the present series cannot assess treatment efficacy, and treatment information is presented descriptively. Larger collaborative studies will be required to determine whether specific therapeutic approaches confer benefit in UCOGC. Precision oncology considerations also deserve attention. BRCA2 alterations were identified in two tumors; however, matched germline testing was not performed, and their germline versus somatic status could not be established from this study. Where clinically appropriate, such findings may warrant confirmatory germline assessment and may have potential therapeutic implications. *BRCA2* alterations in prior reports suggest that selected patients may benefit from platinum-based therapy or PARP inhibitor strategies, although this remains unproven in UCOGC specifically [34]. Similarly, case reports of durable responses to immune checkpoint blockade in metastatic or treatment-refractory pancreatic carcinoma with UCOGC features suggest that immunotherapy may be relevant in selected molecular or immune contexts, particularly when tumor mutational burden, mismatch repair status, PD-L1 expression, or other biomarkers are favorable [35]. These reports should not be overgeneralized, but they strengthen the rationale for comprehensive molecular and immune profiling in this rare tumor.

This study has limitations inherent to research on rare tumors. The small cohort precluded statistical modeling and limits conclusions regarding prognosis or treatment response. In addition, the absence of a contemporaneous stage- and treatment-matched conventional PDAC control group precludes direct comparison of survival between UCOGC and PDAC. Retrospective clinical annotation may be incomplete, and treatment regimens were heterogeneous. Archival FFPE material may compromise DNA yield and sequencing depth, potentially masking subclonal alterations. Our targeted 152-gene sequencing approach was designed to identify clinically and biologically relevant somatic alterations but was not intended to comprehensively characterize the mechanisms underlying phenotypic divergence between UCOGC and PDAC. We did not systematically evaluate structural variants, complex copy-number alterations, epigenetic changes, or transcriptional programs. Therefore, the paired molecular findings support a shared clonal relationship but do not establish the molecular mechanisms responsible for the distinct UCOGC phenotype. Although paired components shared concordant pathogenic variants, differences in variant allele frequency were observed in some cases. Because tumor purity and cellular composition were not quantitatively standardized between macro-dissected regions, these VAF differences cannot be reliably interpreted as evidence of differences in clonal abundance. Broader multi-region genomic and multi-omic studies will be required to investigate these mechanisms and intratumoral heterogeneity.

## 5. Conclusions

In summary, our findings demonstrate shared molecular alterations between morphologically distinct UCOGC and associated ductal components, providing additional evidence supporting a common clonal origin. Clinical outcomes varied considerably within this small cohort; however, given the limited sample size and absence of a matched PDAC comparator, these observations should be considered descriptive and hypothesis-generating. The principal contribution of this series is the integration of detailed morphology, immunophenotyping, component-specific molecular analysis, and clinical annotation in this rare pancreatic tumor. Larger multi-institutional studies incorporating broader genomic, transcriptomic, epigenetic, and microenvironmental analyses will be necessary to clarify the biological basis of the UCOGC phenotype and identify reliable prognostic and therapeutic biomarkers.

## Figures and Tables

**Table 1:** Clinicopathologic Characteristics, Treatment, Molecular Profiles, and Outcomes of Six Pancreatic UCOGC Cases.

Case	Age/Sex	Site	Stage	Surgery	Neoadjuvant Therapy	Adjuvant/Systemic Therapy	NGS in Sarcomatous/Epithelial Component	OS (Months)
1	72/F	Pancreatic neck	T1N0	Yes	No	Gemcitabine/capecitabine	KRAS, TP53/PDAC component unavailable for testing	78
2	63/F	Head	T3N1	Yes	FOLFIRINOX *	Patient declined further therapy	SMAD4, TP53/NGS failed	4
3	60/F	Tail	T3N1M1	Yes	No	FOLFIRINOX	KRAS, TP53/KRAS, TP53 (concordant)	71
4	64/F	Head	T2N0	Yes	No	Gemcitabine/capecitabine	KRAS, TP53/KRAS, TP53 (concordant)	56
5	74/M	Body/tail	T3N0	Yes	No	FOLFIRINOX	BRCA2, CDKN2A/B loss/BRCA2, CDKN2A/B loss (concordant)	17
6	73/M	Head/body	T2N1	Yes	FOLFIRINOX	FOLFIRINOX	KRAS, TP53/KRAS, TP53 (concordant)	1

* FOLFIRINOX: folinic acid (leucovorin), fluorouracil (5-FU), irinotecan, and oxaliplatin; NGS, next-generation sequencing; PDAC, pancreatic ductal adenocarcinoma; OS, overall survival.

## Data Availability

The data supporting the findings of this study are provided within the article and its Supplementary Table S1.

## Supplementary Materials

The following supporting information can be downloaded at: https://www.mdpi.com/article/10.3390/cancers18182967/s1, Table S1: Complete Targeted Next-Generation Sequencing Results by Case and Tumor Component.
