# Supplementary material for: Beyond Morphology: Molecular Evidence Supporting a Clonal Relationship Between Pancreatic Undifferentiated Carcinoma with Osteoclast-like Giant Cells (UCOGC) and Conventional Pancreatic Ductal Adenocarcinoma (PDAC)
Source: Cancers (Basel). 2026 Sep 14;18(18):2967. doi: 10.3390/cancers18182967 (PMC13604581; doi:10.3390/cancers18182967)
Supplement: Supplementary file 1 [file cancers-18-02967-s001.zip › cancers-4543116-supplementary.pdf]

Supplementary Table S1. Complete Targeted Next-Generation Sequencing Results by Case and Tumor Component

Variants are listed by case and separately macro-dissected tumor component. Tier I/II variants indicate variants of strong or potential clinical significance; Tier III indicates variants of uncertain significance.

Case	Gene	Genomic position (hg19)	RefSeq transcript	CDS change	Protein change	COSMIC ID	Read coverage	VAF (%)	Tier	Component	Notes
1	**KRAS**	chr12:25398284	NM_004985.3	c.35G>T	p.Gly12Val	520	272	34	I/II	*Sarcomatoid*	No adenocarcinoma component available for testing
1	**TP53**	chr17:7579336	NM_000546.5	c.336_351del	p.Phe113GlnfsTer5		382	53	I/II	*Sarcomatoid*	
1	**ELP1**	chr9:111644410	NM_003640.3	c.3280A>G	p.Arg1094Gly		196	56	III	*Sarcomatoid*	
1	**JAK1**	chr1:65312368	NM_002227.2	c.1951G>A	p.Val651Met	6930134	243	43	III	*Sarcomatoid*	
1	**STK11**	chr19:1220703	NM_000455.4	c.721G>A	p.Ala241Thr	6419312	229	56	III	*Sarcomatoid*	
2	**APC**	chr5:112173848	NM_000038.5	c.2557G>T	p.Glu853Ter	18566	272	10	I/II	*Sarcomatoid*	Adenocarcinoma component failed testing
2	**APC**	chr5:112175951	NM_000038.5	c.4666dup	p.Thr1556AsnfsTer3	18561	310	32	I/II	*Sarcomatoid*	
2	**BRAF**	chr7:140453136	NM_004333.4	c.1799T>A	p.Val600Glu	476	248	15	I/II	*Sarcomatoid*	
2	**BRCA2**	chr13:32911298	NM_000059.3	c.2808_2811del	p.Ala938ProfsTer21	219056	401	23	I/II	*Sarcomatoid*	
2	**PIK3CA**	chr3:178928067	NM_006218.2	c.1345C>A	p.Pro449Thr	18601	415	31	I/II	*Sarcomatoid*	
2	**SMAD4**	chr18:48586262	NM_005359.5	c.931C>T	p.Gln311Ter		366	29	I/II	*Sarcomatoid*	
2	**TP53**	chr17:7577120	NM_000546.5	c.818G>A	p.Arg273His	10660	145	15	I/II	*Sarcomatoid*	
2	**AXIN1**	chr16:347209	NM_003502.3	c.1802C>T	p.Ala601Val		142	16	III	*Sarcomatoid*	
2	**BRAF**	chr7:140534557	NM_004333.4	c.356C>G	p.Thr119Ser	2861463	328	33	III	*Sarcomatoid*	
2	**SIX1**	chr14:61115363	NM_005982.3	c.545C>T	p.Ala182Val	4621599	223	9	III	*Sarcomatoid*	
2	**TCF3**	chr19:1625622	NM_003200.3	c.452C>T	p.Pro151Leu	4621631	113	12	III	*Sarcomatoid*	
3	**KRAS**	chr12:25398284	NM_004985.3	c.35G>T	p.Gly12Val	520	245	27	I/II	*Adenocarcinoma*	
3	**TP53**	chr17:7579470	NM_000546.5	c.216_217ins	p.Val73ArgfsTer76		188	27	I/II	*Adenocarcinoma*	
3	**LYST**	chr1:235827874	NM_000081.3	c.11086G>A	p.Val3696Ile		206	43	III	*Adenocarcinoma*	
3	**SMO**	chr7:128845511	NM_005631.4	c.808G>A	p.Val270Ile	1738148	251	46	III	*Adenocarcinoma*	
3	**KRAS**	chr12:25398284	NM_004985.3	c.35G>T	p.Gly12Val	520	241	20	I/II	*Sarcomatoid*	
3	**TP53**	chr17:7579470	NM_000546.5	c.216_217ins	p.Val73ArgfsTer76		155	6	I/II	*Sarcomatoid*	
3	**LYST**	chr1:235827874	NM_000081.3	c.11086G>A	p.Val3696Ile		232	52	III	*Sarcomatoid*	
3	**SMO**	chr7:128845511	NM_005631.4	c.808G>A	p.Val270Ile	1738148	235	43	III	*Sarcomatoid*	
4	**KRAS**	chr12:25398284	NM_004985.3	c.35G>A	p.Gly12Asp	521	632	30	I/II	*Adenocarcinoma*	
4	**TP53**	chr17:7578553	NM_000546.5	c.377A>G	p.Tyr126Cys	11517	281	15	I/II	*Adenocarcinoma*	
4	**RET**	chr10:43608354	NM_020975.4	c.1702G>A	p.Gly568Ser	7455374	396	40	III	*Adenocarcinoma*	
4	**KRAS**	chr12:25398284	NM_004985.3	c.35G>A	p.Gly12Asp	521	555	6	I/II	*Sarcomatoid*	
4	**TP53**	chr17:7578553	NM_000546.5	c.377A>G	p.Tyr126Cys	11517	424	2	I/II	*Sarcomatoid*	
4	**RET**	chr10:43608354	NM_020975.4	c.1702G>A	p.Gly568Ser	7455374	401	43	III	*Sarcomatoid*	
5	**BRCA2**	chr13:32913558	NM_000059.3	c.5073dup	p.Trp1692MetfsTer3	5078974	547	51	I/II	*Adenocarcinoma*	
5	**CDKN2A/B loss**	—	—	—	—	—	—	—	I/II	*Adenocarcinoma*	
5	**BRCA2**	chr13:32913558	NM_000059.3	c.5073dup	p.Trp1692MetfsTer3	5078974	351	53	I/II	*Sarcomatoid*	

Case	Gene	Genomic position (hg19)	RefSeq transcript	CDS change	Protein change	COSMIC ID	Read coverage	VAF (%)	Tier	Component	Notes
5	**CDKN2A/B loss**	—	—	—	—	—	—	—	I/II	*Sarcomatoid*	
6	**GNAS**	chr20:57484420	NM_000516.4	c.601C>T	p.Arg201Cys	27887	764	44	I/II	*Adenocarcinoma*	
6	**KRAS**	chr12:25398284	NM_004985.3	c.35G>A	p.Gly12Asp	521	841	61	I/II	*Adenocarcinoma*	
6	**RB1**	chr13:48954351	NM_000321.2	c.1472_1473del	p.Leu491ArgfsTer8		443	45	I/II	*Adenocarcinoma*	
6	**TP53**	chr17:7579530	NM_000546.5	c.156dup	p.Trp53MetfsTer4		231	50	I/II	*Adenocarcinoma*	
6	**RET**	chr10:43622132	NM_020975.4	c.3149G>A	p.Arg1050Gln	7932533	405	49	III	*Adenocarcinoma*	
6	**SMO**	chr7:128851867	NM_005631.4	c.1939C>T	p.Pro647Ser	9154673	217	40	III	*Adenocarcinoma*	
6	**GNAS**	chr20:57484420	NM_000516.4	c.601C>T	p.Arg201Cys	27887	986	44	I/II	*Sarcomatoid*	
6	**KRAS**	chr12:25398284	NM_004985.3	c.35G>A	p.Gly12Asp	521	1045	56	I/II	*Sarcomatoid*	
6	**RB1**	chr13:48954351	NM_000321.2	c.1472_1473del	p.Leu491ArgfsTer8		647	53	I/II	*Sarcomatoid*	
6	**TP53**	chr17:7579530	NM_000546.5	c.156dup	p.Trp53MetfsTer4		361	44	I/II	*Sarcomatoid*	
6	**RET**	chr10:43622132	NM_020975.4	c.3149G>A	p.Arg1050Gln	7932533	522	46	III	*Sarcomatoid*	
6	**SMO**	chr7:128851867	NM_005631.4	c.1939C>T	p.Pro647Ser	9154673	347	52	III	*Sarcomatoid*	

Abbreviations: CDS, coding DNA sequence; VAF, variant allele frequency; NGS, next-generation sequencing.

VAF is shown as a percentage. Read coverage and VAF are not applicable to copy-number alterations such as CDKN2A/B loss.

Case 1: no adenocarcinoma component was available for molecular testing. Case 2: testing of the adenocarcinoma component failed.

Tier classification follows the AMP/ASCO/CAP four-tier framework (PMID: 27993330).
